# Fibroblast and Lymphoblast Gene Expression Profiles in Schizophrenia: Are Non-Neural Cells Informative?

**DOI:** 10.1371/journal.pone.0002412

**Published:** 2008-06-11

**Authors:** Nicholas A. Matigian, Richard D. McCurdy, François Féron, Christopher Perry, Heather Smith, Cheryl Filippich, Duncan McLean, John McGrath, Alan Mackay-Sim, Bryan Mowry, Nicholas K. Hayward

**Affiliations:** 1 Queensland Institute of Medical Research, Herston, Queensland, Australia; 2 Queensland Centre for Mental Health Research, Wacol, Queensland, Australia; 3 Sick Kids Research Institute, Toronto, Ontario, Canada; 4 Neurobiologie des Interactions Cellulaires et Neurophysiopathologie, CNRS UMR 6184. Bd Pierre Dramard, Marseille, France; 5 Department of Surgery, University of Queensland, St Lucia, Queensland, Australia; 6 Eskitis Institute for Cell and Molecular Therapies, Griffith University, Nathan, Queensland, Australia; 7 Department of Psychiatry, University of Queensland, St Lucia, Queensland, Australia; James Cook University, Australia

## Abstract

Lymphoblastoid cell lines (LCLs) and fibroblasts provide conveniently derived non-neuronal samples in which to investigate the aetiology of schizophrenia (SZ) using gene expression profiling. This assumes that heritable mechanisms associated with risk of SZ have systemic effects and result in changes to gene expression in all tissues. The broad aim of this and other similar studies is that comparison of the transcriptomes of non-neuronal tissues from SZ patients and healthy controls may identify gene/pathway dysregulation underpinning the neurobiological defects associated with SZ. Using microarrays consisting of 18,664 probes we compared gene expression profiles of LCLs from SZ cases and healthy controls. To identify robust associations with SZ that were not patient or tissue specific, we also examined fibroblasts from an independent series of SZ cases and controls using the same microarrays. In both tissue types ANOVA analysis returned approximately the number of differentially expressed genes expected by chance. No genes were significantly differentially expressed in either tissue when corrected for multiple testing. Even using relaxed parameters (p≤0.05, without multiple testing correction) there were still no differentially expressed genes that also displayed ≥2-fold change between the groups of SZ cases and controls common to both LCLs and fibroblasts. We conclude that despite encouraging data from previous microarray studies assessing non-neural tissues, the lack of a convergent set of differentially expressed genes associated with SZ using fibroblasts and LCLs indicates the utility of non-neuronal tissues for detection of gene expression differences and/or pathways associated with SZ remains to be demonstrated.

## Introduction

As with many complex clinical phenotypes, the identification of heritable factors associated with schizophrenia (SZ) remains a challenge [1]. Despite a lack of agreement in exact details of polymorphisms associated with SZ between different populations, there is optimism that data from gene association studies, together with analyses of gene expression studies in post-mortem brain, can provide convergent evidence of candidate biological pathways associated with the phenotype. For example, Harrison and Weinberger have noted that many of the candidate susceptibility genes associated with SZ are associated with synaptic plasticity [2]. The goal is to identify the dysregulated neurobiological pathways that are associated with the genetic, cellular and developmental pathways that result in the manifestation of SZ. High-throughput microarray gene expression profiling is an effective approach for the identification of candidate genes and associated molecular pathways implicated in a wide variety of biological processes or disease states. To date there has been a lack of consistency with respect to the individual genes identified in gene expression studies based on comparison of post-mortem brain tissue from SZ cases and healthy controls. However, there has been a degree of concordance in some gene ontology categories/pathways proposed to characterise SZ. These include: increased expression of genes involved in pre-synaptic function [3]–[6]; down-regulation of genes involved in energy production [7]–[9], and down-regulation of myelination/oligodendrocyte related genes [10]–[13]. There are several challenges in generating and replicating expression findings in post-mortem brain: (i) choice of appropriate brain region for investigation; (ii) the heterogeneity of cell types within brain tissue; (iii) the reliance on relatively small samples; and (iv) the impact of cause of death and/or post-death handling of tissue on gene expression [14]. Thus, the use of post-mortem brain tissue, compounded by a range of other factors that contribute to between-study variation (e.g. age, race, gender, different microarray platforms and analysis methods), could underpin the relative lack of gene/transcript-level consistency between expression studies [15]–[18].

To overcome some of these problems several groups have considered the use of samples other than post-mortem brain. For instance, lymphocytes [19] and fibroblasts [20]–[22]. have been reported to show biological differences between individuals with SZ and healthy controls. The biological pathways themselves (cell-cell signalling, cellular proliferation and death, immune response) have been previously suspected to be involved in the pathophysiology of SZ [23], [24]. In one comparison study, the expression profiles of different tissues from the central nervous system (CNS) were shown to have the highest degree of similarity to expression profiles from whole blood compared to any other tissue type [25]. Notably, of 45 genes previously implicated in the aetiology of SZ, 21 were expressed both in whole blood and the CNS. Recently, non-brain cell sources have emerged as an alternative for investigating gene expression differences in SZ, including: peripheral blood leukocytes (PBLs) [26], [27]; lymphoblastoid cell lines (LCLs) [28], [29]; and olfactory epithelium (OE) [30]. The results, to date, have shown little overlap with gene array studies based on post-mortem brain tissue. In one study comparing gene expression in different tissues, Glatt et al [31] assessed expression of prefrontal cortex (PFC) post-mortem brain and peripheral blood cells from different cohorts of SZ patients and controls, in order to identify genes with differential expression across populations and tissue types. They applied rigorous statistical analyses to limit false positives and found six genes that were differentially expressed in both tissues - less than expected by chance. Only one of these genes (*SELENBP1*) displayed the same direction of change, the differential expression of which was confirmed in lymphocytes by qRT-PCR and in brain via immunohistochemistry.

The use of these alternative tissues is relevant to psychiatric disease researchers because blood-based tissues (lymphocytes) are more readily obtained, thereby allowing larger case-control studies with optimal matching on key variables (e.g. age, race, sex). However, the underlying hypothesis of such studies is the existence of subtle, disease-related effects in all tissues of the body, but which only exert a detrimental/disease-causing effect in the brain. This hypothesis warrants validation – while non-neuronal tissues are clearly convenient alternatives to post-mortem brain, questions remain as to how informative gene expression studies based on these samples will be. For example, if disease-related effects impact on tissues other than the brain, one would predict that gene expression studies based on two different tissue types should provide comparable patterns of dysregulation.

The aim of this study was to compare gene expression in two different non-neural tissue sources (LCLs and fibroblasts) from patients with SZ versus controls in order to detect a robust set of common SZ-associated defects in both tissues.

## Materials and Methods

### Participants

#### Lymphocytes

Participants in this study were recruited as part of the Brisbane Psychosis Study (BPS), a case-control study, the full details of which are provided elsewhere [32]. Samples were well matched for age and gender distribution and in total 8 SZ samples and 7 control samples, recruited from the general population of Brisbane, were used in this study (Table 1). All subjects were assessed with a modified Schedule for Clinical Assessment in Neuropsychiatry and each patient diagnosis/well control status was confirmed with a computerised diagnostic system (OPCRIT). The diagnosis of SZ was assigned according to DSM-IIIR criteria [33]. All subjects included in this study provided written, informed consent and the study was approved by the Wolston Park Hospital Institutional Ethics Committee.

**Table 1:** Information on schizophrenia patient and control samples.

Sample ID	Sex	Age	Antipsychotic Medication
**LCL Samples**			
**SZ patients**			
S1L	M	29	None
S2L	M	21	Clozapine
S3L	M	26	Clozapine
S4L	M	20	Clozapine
S5L	M	23	Clozapine
S6L	M	24	Risperidone
S7L	M	26	Clozapine
S8L	M	22	Clozapine
**Controls**			
C1L	M	20	None
C2L	M	26	None
C3L	M	23	None
C4L	M	21	None
C5L	M	25	None
C6L	M	27	None
C7L	M	26	None
**Fibroblast Samples**			
**SZ patients**			
S1F	F	46	Pericyazine
S2F	M	23	Zuclopenthixol
S3F	M	30	Olanzapine
S4F	F	44	Clozapine
S5F	M	32	Trifluoperazine
S6F	M	50	Olanzapine
S7F	M	38	Flupenthixol; Olanzapine
S8F	F	44	Risperidone
**Controls**			
C1F	M	23	None
C2F	M	41	None
C3F	F	29	None
C4F	M	45	None
C5F	M	25	None
C6F	M	53	None
C7F	F	29	None

#### Fibroblasts

Patients and controls in this aspect of the study were also recruited as part of a study examining OE cultures in psychosis [30]. A total of 8 SZ samples and 7 control samples were used in this study (Table 1).

### Sample preparation

#### Lymphoblastoid cell lines

LCLs were established by Epstein-Barr virus transformation of lymphocytes as described [34]. For RNA, cell lines were all grown under tightly controlled growth conditions in the same batch of RPMI 1640 medium with 10% FCS and antibiotics, to limit variation in RNA production related to cell culturing effects. Total RNA was extracted when cells were in log phase growth and had been cultured for approximately the same number of passages (approx. 4–6). RNA from all samples was run on an Agilent Bioanalyzer to assure quality and to obtain concentration.

#### Fibroblasts

Skin biopsies (1 mm^2^) were collected under local anaesthesia from the upper arm using aseptic conditions and immediately immersed in 2 ml Dulbecco's Modified Eagles Medium (DMEM). Primary cultures were established using the DMEM growth medium, with the modification of 1% benzylpenicillin/streptomycin sulphate. Biopsies were carefully placed in Petri-dishes (60 mm^2^) and covered with a coverslip to prevent air-bubble formation. DMEM growth medium (600 µl) was added and explants incubated under optimal growth conditions (37°C, 5% CO_2_). Epithelial outgrowth was monitored under a microscope at 24 hour intervals, and DMEM growth medium was supplemented with 100 µl fresh medium after 24 hours initially, and subsequently every 48 hours. When fibroblast outgrowth reached confluency in the Petri-dish, the fibroblasts were redistributed into 25 cm^2^ tissue culture flasks. When these cells reached confluency, they were pelleted and stored in liquid nitrogen

Fibroblast cultures were re-established by rapidly thawing the sample at 37°C and transferred to a tissue culture flask. Cell growth was monitored every 24 hours and RPMI 1640 growth media replaced every 48 hours. When fibroblast outgrowth reached confluency cells were split into larger tissue culture flasks until enough cells were obtained for a RNA extraction (six flasks, each approximately 2–4×10^7^ cells). RNA extraction was undertaken using QIAGEN RNeasy® midi-columns (Qiagen, Clifton Hill, Victoria) using the animal cell protocol as outlined in the product manual. RNA from all samples was run on an Agilent Bioanalyzer to assess quality and to determine concentration.

### RNA labelling and microarray hybridisation

A common reference experimental design was used. Human universal reference from Stratagene was used as the reference RNA, and labelled with Cyanine 3 dye in all microarray experiments. The experiments were performed on Human Genome v.2.1 oligo arrays, available from the Gene Array Facility at The Prostate Centre, Vancouver General Hospital (http://prostatelab.org/arraycentre/index.html). Each array contained 18,664 unique cDNA elements. Total RNA was used to generate fluorescently labelled cDNA by the indirect amino allyl dUTP (AA-dUTP) method using the Superscript™ ΙΙΙ Reverse Transcriptase System. This is a two-step method; in the first step amino allyl dUTP, an amine-modified nucleotide, is incorporated during reverse transcription. Subsequently, monofunctional forms of Cyanine 3 (Cy3) and Cyanine 5 (Cy5) dyes are reacted with the AA-dUTP labelled cDNA. The labelled cDNA was purified and incubated on an array at 37°C for 14 hours. Following hybridization, slides were washed and scanned using an Affymetrix/Genetic Microsystems 418 Array Scanner (Genetic Microsystems). Data capture was performed using the software ImaGene™ Version 5.1 (BioDiscovery, California, USA).

### Data analysis and filtering

The pixel intensity data from ImaGene™ were imported into GeneSpring™ 7.2 (Agilent Technologies, CA) and signal intensities for each spot, sample and reference signals were corrected for background, normalised for intensity (Lowess residual), and a ratio generated. The data was centralized by dividing each measurement by the 50th percentile of all measurements in that sample, to control for chip-wide variations in intensity. Quality control data filtering was then performed to remove signals that were present in <85% of samples (<13/15) and with expression values below that of the background as calculated by the cross-gene error model [35].

When using LCLs (virus-transformed B-lymphocytes), different subclones of B-cells could be randomly (a) infected with EBV, and (b) selected in culture. Consequently, all immunoglobulin and B-cell-related genes were removed from analysis, because any apparent differences in the expression of these genes are more likely to be an artifact rather than due to the disease under investigation [28], [36]. Differential expression was determined by one-way ANOVA-Welch's approximate t-test. A p-value cut off <0.05 was used for the mean difference between groups. Additionally a 1.2-fold change filter was imposed on the genes found to be differentially expressed in SZ LCLs in order to compare our data directly with those of Vawter and colleagues [29]. who also assessed gene expression in SZ versus control LCLs. The GenBank Accession numbers were used to detect any common probes/genes between the two LCL gene lists.

## Results

### Lymphoblastoid cell lines

After quality control filtering and removal of immune/B-cell related probes, 8,500 transcripts remained. Differential expression was determined using the Welch t-test, a p-value of <0.05 returned 550 probes. Of these, 545 and 10 had fold-changes of ≥1.2 and ≥2 respectively, between the means of the SZ and control groups. When p-values were adjusted for multiple comparisons, there were no genes that remained significantly differentially expressed (Table 2).

**Table 2:** Results of quality control, statistical, fold-change and overlap filtering.

	number of genes in LCLs	number of genes in fibroblasts	number of genes in common
Filtered 85%^a^	8500	9999	5442
Expected by Chance 0.05	425	500	-
Welch 0.05	550	434	15
Welch 0.05+B&H FDR^b^	0	0	0
Welch 0.05+2 fold	10	0	0
Welch 0.05+1.2 fold^c^	545	339	2
Welch 0.05+1.2 fold Up	238	189	0
Welch 0.05+1.2 fold Down	307	150	2

a Data were filtered to have signal above background in ≥13/15 samples

b B&H FDR - Benjamini and Hochberg False Discovery Rate multiple testing correction

c This statistical and fold-change cut-off was chosen so data could be compared to Vawter et al [29]

### Fibroblasts

Out of the 9,999 transcripts which remained after quality control filtering, 434 transcripts were determined to be differentially expressed (Welch t-test, p<0.05). Of these, 339 and 0 had fold-changes of ≥1.2 and ≥2 respectively, between the means of the SZ and control groups. Correction for multiple testing returned no significantly differentially expressed genes (Table 2).

### Lymphoblast and fibroblast overlap

There were 15 probes in common between the LCL and fibroblast differentially expressed probe lists when no fold-change cut-off was applied or direction of change considered. However, when direction and 1.2 fold-change filtering were imposed, only 2 of these genes (*ADSL* [-1.50-fold in LCLs and -1.28-fold in fibroblasts, in SZ relative to controls] and *LOC441204* [-1.24-fold in LCLs and -1.23-fold in fibroblasts, in SZ compared to controls]) displayed the same direction of dysregulation (Table 2). When only the direction of change was considered, irrespective of the magnitude of the change, 1 additional gene (*FLJ14833*) was putatively differentially expressed in common between fibroblasts (-1.18-fold in SZ) and LCLs (-1.43-fold in SZ) from SZ patients compared with controls.

### Overlap with previously published LCLs expression data

Of 28 differentially expressed genes detected by Vawter et al [29], 27 were represented on the microarrays used in our study. There was only one gene, encoding basic transcription factor 3 (*BTF3*; NM_001207), in common between the 545 statistically significant genes (*p*<0.05) with a ≥1.2 fold-change detected in LCLs is this study and the 27 genes reported by Vawter et al [29]. This number of genes in common is that expected by random chance.

## Discussion

In this study we could find no constitutive gene expression differences of large effect between unrelated SZ cases and controls in either LCLs or fibroblasts. When a multiple testing correction was applied there were no significantly differentially expressed genes between SZ and controls in either tissue type. Moreover, we also failed to find a significant overlap between the list of differentially expressed genes in our panel of LCLs and those reported by Vawter et al [29]. in a similar analysis. Although our study is of limited sample size, we would generally expect small samples to contribute to a high type 1 error rate. However, this was not observed; the number of differentially expressed gene in the case-control comparison of both tissues was approximately that expected by chance. It should be noted that the LCLs and fibroblast cultures were from non-overlapping case-control studies. This adds a randomization to the tissue group comparisons which should act as a filter to identify the more robust changes due to the disease by decreasing noise due to chance differences in heritable gene expression levels between individuals. An alternative strategy would be to conduct between-tissue expression studies based on samples from the same individuals. But because SZ is a heterogeneous group of disorders, either approach may make any true system-wide dysregulation of transcription difficult to detect, especially if the effect size is small. When testing 20,000 genes with a per-gene alpha value of 0.05 the number of samples needed to detect differentially expressed genes by t-tests with a fold change of 1.2 is 112, whereas only eight samples are needed to statistically have the power to detect a 2-fold difference (http://bioinformatics.mdanderson.org/MicroarraySampleSize/MicroarraySampleSize.aspx). By comparison, the LCL and fibroblast data sets used in the current study each had the power to detect a SZ-related expression difference of 1.65-fold. It is entirely feasible that any expression differences related to the aetiology of SZ would be of small effect, and that when testing unrelated individuals, it could be below the level of natural variation.

Moreover, gene expression analysis of case-control samples does not take into account the notion of individual-specific thresholds for disease causality. With this concept, the absolute level of expression of a given gene required to trigger SZ might be different between individuals, and would be ‘set’ by their constitutive global gene expression profile. One approach to overcome this potential limitation is the use of monozygotic twins discordant for disease. This type of analysis provides a powerful tool for reducing genetic variation between cases and controls, while simultaneously enhancing the detection of epigenetic differences emanating from in utero or post-natal environmental exposures [37]. We, and others, have used this approach successfully in the study of gene expression differences in bipolar disorder [28], [36] and autism [38]. Thus its application to SZ seems warranted.

Although the two non-neuronal tissues we have tested here have provided limited utility in identifying SZ-associated gene expression abnormalities, it is plausible that very large case-control samples (to reduce individual variation/SZ heterogeneity) of LCLs or fibroblasts may be suitable for validation (e.g. by qRT-PCR) of a small number of genes identified via other means. Additionally, there are other non-post-mortem brain tissue sources that may prove fruitful. For example, olfactory epithelium, a tissue analogous to the neuroepithelium of the neural tube from which the brain develops in the embryo has yet to be fully assessed. Cultures of this tissue have been used to detect differences between disease and non-diseased states [39], [40] and thus could provide a more relevant alternative to tissues of non-neuronal origin, such as fibroblasts and LCLs, for studying a range of neurological disorders. We have reported the findings of one such study comparing gene OE expression profiles between SZ cases and controls [30]. Although it should be noted that this was an exploratory study and the conclusions should be interpreted cautiously due to the heterogeneous cell population from the nasal biopsy and the small amount of material obtained, which did not allow for any validation of gene expression differences. A better alternative still may be to derive neural stem cell lines from the OE and use these to analyze gene expression changes related to SZ and other brain diseases.

The ability to use non-neuronal tissues to explore transcription in neuropsychiatric disorders is of heuristic value, allowing the recruitment of larger sample sizes. While some studies have suggested that white blood cells may be an appropriate alternative to neuronal tissue, based on comparable expression of many relevant genes and pathways [25] the results of the current study do not support the hypothesis. The lack of robust disease-related gene expression differences in both fibroblasts or LCLs weakens the case that these non-neuronal tissue sources are informative for detecting the underlying causative genetic and epigenetic changes responsible for SZ predisposition and development. However, the relatively small sample sizes used here do not rule out the possibility of consistent yet subtle changes in gene expression being detected in case-control analyses of substantially larger cohorts.
